# Estimating the human bottleneck for contact tracing

**DOI:** 10.1093/pnasnexus/pgae283

**Published:** 2024-07-16

**Authors:** Maximilian D Broda, Petra Borovska, Diana Kollenda, Marcel Linka, Naomi de Haas, Samuel de Haas, Benjamin de Haas

**Affiliations:** Experimental Psychology, Justus Liebig University Giessen, Otto-Behaghel-Str 10F, 35394 Giessen, Germany; Center for Mind, Brain and Behavior, Universities of Marburg, Giessen and Darmstadt, Hans-Meerwein-Strasse 6, 35032 Marburg, Germany; Experimental Psychology, Justus Liebig University Giessen, Otto-Behaghel-Str 10F, 35394 Giessen, Germany; Experimental Psychology, Justus Liebig University Giessen, Otto-Behaghel-Str 10F, 35394 Giessen, Germany; Experimental Psychology, Justus Liebig University Giessen, Otto-Behaghel-Str 10F, 35394 Giessen, Germany; Independent researcher; Chair for Industrial Organization, Regulation and Antitrust, Department of Economics, Justus Liebig University Giessen, Licher Straße 62, 35394 Giessen, Germany; Experimental Psychology, Justus Liebig University Giessen, Otto-Behaghel-Str 10F, 35394 Giessen, Germany; Center for Mind, Brain and Behavior, Universities of Marburg, Giessen and Darmstadt, Hans-Meerwein-Strasse 6, 35032 Marburg, Germany

**Keywords:** contact tracing, memory, forgetting, under-reporting

## Abstract

The SARS-CoV-2 pandemic has highlighted the importance of contact tracing for epidemiological mitigation. Contact tracing interviews (CTIs) typically rely on episodic memory, which is prone to decline over time. Here, we provide a quantitative estimate of reporting decline for age- and gender-representative samples from the United Kingdom and Germany, emulating >15,000 CTIs. We find that the number of reported contacts declines as a power function of recall delay and is significantly higher for younger subjects and for those who used memory aids, such as a scheduler. We further find that these factors interact with delay: Older subjects and those who made no use of memory aids have steeper decline functions. These findings can inform epidemiological modeling and policies in the context of infectious diseases.

## Introduction

The SARS-CoV-2 pandemic has highlighted the importance of contact tracing for public health. Epidemiological mitigation can crucially depend on case isolation (1) which requires identification of infected individuals and their chain of contacts (1, 2). This is traditionally attempted by standardized contact tracing interviews (CTIs). Modeling suggested that CTIs suffered from severe speed limitations in the context of SARS-CoV2 (2, 3), leading many nations to implement digital contact tracing apps (CTAs) (4). However, CTAs suffered from limited adoption (5). This has led to the parallel use of traditional CTIs, requiring infected individuals to report contacts going back up to 10 days or more.

A principal bottleneck of CTIs is memory decline (6–10). Basic and applied research has shown that long-term episodic memory is susceptible to distortions, interference and loss of information (6, 8, 11–13). However, it is largely unclear *how* memory decline affects CTIs. What fraction of relevant contacts do people typically remember after a day, a week, or two? Does declining contact memory follow a linear (14), or power function (12, 13) of recall delay? And how is this modulated by age and the use of memory aids, such as a scheduler? Quantitative estimates of this bottleneck could provide lower bounds of under-reporting and thus inform epidemiological modeling and policies.

A major obstacle in the way of quantifying the decline of contact memory is the unknown ground truth, which is expected to vary over time. Ben may have met particularly many people during a birthday party last Saturday, while Max's number of contacts peaked during an office meeting on Wednesday. Pilot data indicate that individual contact reports can indeed vary by orders of magnitude across days.

Here, we aimed to overcome these challenges with the power of large samples. With the help of a panel provider, we recruited 15,015 subjects in April and May 2023, aiming at age- and gender-representative samples of the 18- to 74-year-old populations of the United Kingdom and Germany (GER). Subjects completed an online questionnaire designed in consultation with local health authorities (Gesundheitsamt, Landkreis Giessen, Germany) to emulate CTIs. Specifically, the questionnaire asked subjects to make use of memory aids such as a scheduler or calendar app wherever possible and provided a definition of relevant contacts. Then, subjects were asked to note such contacts on a sheet of paper, count them and enter their number into the survey, separately for increasingly wider domains of social interaction. Every subject repeated this procedure for each of the 14 preceding days in turn (i.e. with increasing reporting delay). Finally, subjects indicated their use of memory aids using a five-point Likert scale. A total of 13,407 interviews were included into the final analysis (6,733 and 6,674 for United Kingdom and GER, respectively; see Supplementary material for details).

We find that the average number of reported contacts indeed is subject to decline, following a power function of reporting delay. The slope and intercept of this decline interacts with age and the use of memory aids, indicating more severe contact memory decline for older subjects and those who reported no use of memory aids.

**Fig. 1.**
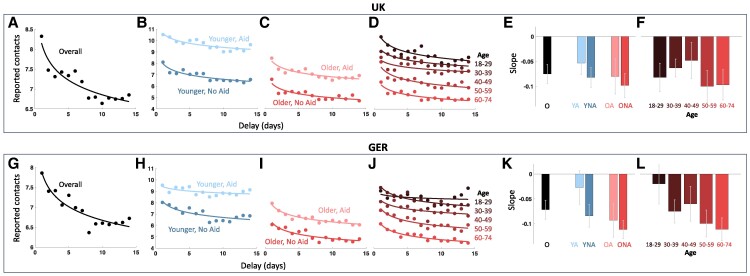
Memory decline for the United Kingdom and GER samples. Scatter points in (A–D) and (G–J) show the average number of reported contacts for 1–14 days of reporting delay. The corresponding line fits show the best fitting power functions. A and G) show the data for all respective subjects, B, C, H, and I) split the data according to age and aid use, indicated by hue and saturation as labeled. D and J) split the data into finer age brackets as indicated by hue, as labeled. Bars in (E, F) and (K, L) show the best fitting slopes of the corresponding power functions. Gray error bars indicate 95% confidence intervals. O, overall; YA, younger subjects using aids; YNA, younger subjects using no aids; OA, older subjects using aids; ONA, older subjects using no aids.

First, we computed the average number of reported contacts, separately for each reporting delay and sample (Fig. 1A and G). In both samples, reported contacts declined with delay. This relationship was captured well by power functions of the form


(1) $$\mathrm{AC}=\;b\;*\;\mathrm{dela}y^{m},$$

with AC corresponding to the average number of reported contacts, delay to the reporting *delay* in days, and factor *b* and exponent *m* to intercept and slope parameters, respectively. For the United Kingdom and GER, these power fits explained 85 and 84% of variance and had significantly negative exponents of −0.075 (95% CI: −0.095, −0.056) and −0.072 (95% CI −0.091, −0.053), respectively (black bars in Fig. 1E and K). The power decline functions provided a better explanation of the data than linear fits, as indicated by the Akaike Information Criterion (ΔAIC < −8.7 for both samples).

Next, we split the data according to median age (United Kingdom: 45, GER: 47) and the reported use of memory aids, such as a scheduler (55%/51% of United Kingdom/GER subjects reported no aid use, see Supplementary material; Fig. 1B, C, H, and I). We found moderate to strong evidence in favor of power over linear fits for each of the four resulting subdivisions (Table 1). As can be seen in Fig. 1, the overall number of reported contacts was substantially lower for older compared to younger subjects and for subjects who reported no use of memory aids compared to those who did. In addition to these intercept effects, the best fitting slope parameters indicated a steeper decline of reported contacts for older subjects and those who reported no use of memory aids (Fig. 1E and K; Table 1).

**Table 1.** Parameters (with 95% confidence intervals [CI]) of best fitting power functions for the daily means of reported contacts in the United Kingdom and GER samples and corresponding subgroups.

	Overall	YA	YNA	OA	ONA
**United Kingdom**					
Intercept (95% CI)	8.15 (7.86, 8.45)	10.62 (10.22, 11.03)	7.97 (7.63, 8.31)	8.19 (7.81, 8.56)	6.23 (5.82, 6.64)
Slope (95% CI)	−0.075 (−0.095, −0.056)	−0.053 (−0.073, −0.033)	−0.082 (−0.105, −0.059)	−0.080 (−0.104, −0.056)	−0.098 (−0.134, −0.063)
*R* ^2^	85%	73%	83%	80%	74%
ΔAIC	−9.12	−2.27	−5.38	−8.92	−7.52
**GER**					
Intercept (95% CI)	7.87 (7.59, 8.15)	9.42 (9.01, 9.83)	8.13 (7.62, 8.64)	7.80 (7.54, 8.07)	6.25 (5.86, 6.65)
Slope (95% CI)	−0.072 (−0.091, −0.053)	−0.027 (−0.050, −0.005)	−0.084 (−0.118, −0.050)	−0.093 (−0.111, −0.075)	−0.112 (−0.146, −0.077)
*R* ^2^ (%)	84	36	70	91	80
ΔAIC	−8.72	−2.22	−4.91	−14.66	−2.51

Intercept and slope parameters correspond to the factor and exponent in Eq. 1. *R*^2^: proportion of variance explained.

ΔAIC, difference in Akaike information criterion compared to best linear fit; O, overall; YA, younger subjects using aids; YNA, younger subjects using no aids; OA, older subjects using aids; ONA, older subjects using no aids.

To test the statistical significance of these effects we entered the full datasets (not just the daily averages) into a Generalized Linear Mixed model (GLMM) of the form:


(2) C∼1+logdelay*Aid+logdelay*Older+(1|Subject)

with a Poisson link function. That is, we modeled the number of reported contacts (*C*) as a function of the delay in days (log transformed to compensate for the nonlinearity of the memory decline), with additional binary fixed effects for aid and age group, as well as fixed effects for their interactions with delay, and a random effect for subject identity. All fixed effects (including the interaction terms) were highly significant, for both the United Kingdom (all |*t*| > 6, all *P* < 10^−9^) and GER (all |*t*| > 12, all *P* < 10^−9^) (Table 2). An additional GLMM splitting the age predictor into five brackets corroborated these results for both the United Kingdom (all |*t*| > 4, all *P* < 10^−5^) and GER (all |*t*| > 8, all *P* < 10^−9^). Fitting separate decline functions for each age bracket showed decreasing intercepts and trends for increasingly negative slopes (Fig. 1D, F, J, and L).

**Table 2.** Predictor weights and statistics for fixed effects in GLME models of reported contacts in the United Kingdom and GER samples (using a Poisson link function).

	Beta (95% CI)	*t*	*P*
**United Kingdom (DF: 94256)**			
Intercept	1.723 (1.684, 1.762)	85.96	<10^−9^
Delay [log(days)]	−0.079 (−0.085, −0.074)	−27.93	<10^−9^
Aid use	0.348 (0.302, 0.393)	15.06	<10^−9^
Older	−0.292 (−0.337, −0.247)	−12.69	<10^−9^
Delay:Aid use	0.024 (0.018, 0.030)	7.42	<10^−9^
Delay:Older	−0.020 (−0.027, −0.014)	−6.22	<10^−9^
**GER (DF: 93430)**			
Intercept	1.747 (1.708, 1.786)	86.94	<10^−9^
Delay [log(days)]	−0.075 (−0.080, −0.069)	−25.46	<10^−9^
Aid use	0.252 (0.207, 0.296)	11.18	<10^−9^
Older	−0.293 (−0.337, −0.249)	−13.03	<10^−9^
Delay:Aid use	0.041 (0.035, 0.048)	12.55	<10^−9^
Delay:Older	−0.048 (−0.055, −0.042)	−14.53	<10^−9^

CI, confidence intervals. Delay was log-transformed to compensate for the nonlinearity. Aid use and Older were entered as binary grouping variables, Delay:Aid use and Delay:Older denote interaction terms. DF, degrees of freedom (apply to all fixed effects).

Taken together, these data provide strong evidence that contact reports in a CTI design used by practitioners decline as a power function of reporting delay. This underscores the substantial cognitive bottleneck for CTIs (6–8) and severity of underreporting even for minor delays. Contact tracing guidelines should utilize recently investigated strategies to mitigate underreporting and speed up the CTI process (6–8, 10, 15). Epidemiological models should take under-reporting, its dependence on delay and interactions with infection dynamics into account.

We found steeper declines of reported contacts for older subjects and those who reported no use of memory aids. The effects of age and aid use were substantial from the first day. This may partly reflect a smaller number of actual contacts for older participants and those who reported no use memory aids. But the interaction with delay clearly indicates more severe underreporting in these subjects. The strong effect of memory aids and frequent lack of their use suggests that CTI designs could profit from routine instructions on how to leverage prevalent memory aids (such as smartphones). This is in line with previous research showing that the design of CTIs can strongly influence the number of reported contacts (6, 7, 10, 15), including recent results showing that the relationship between the number of reported contacts and reporting delay is modulated by the direction of recall (15). Further research is needed to understand the different mechanisms at play and to optimize the design of CTIs.

Reassuringly, all our findings closely replicated across the United Kingdom and GER samples. Nevertheless, our estimates of memory decline may not readily generalize to populations with symptoms or different patterns of social contacts, or to lock-down situations. Further, our design is insensitive to the absolute intercept of under-reporting (which may be exacerbated by early forgetting, carelessness or dishonesty). We therefore consider our estimates lower bounds of under-reporting for the type of CTI design we tested.

## Materials and methods

The study was approved by the institutional review board of FB06, Justus-Liebig University Giessen, and all subjects provided written informed consent. See Supplementary material and Supplementary Appendices for survey details.

## Supplementary Material

pgae283_Supplementary_Data

## Data Availability

Data and code to reproduce all analyses are freely available at: https://osf.io/pkqgb.

## Supplementary Material

Supplementary materialis available at *PNAS Nexus* online.

## Preprints

An earlier version of this manuscript was posted on a preprint server: https://doi.org/10.23668/psycharchives.14175.
