# Supplementary material for: Estimating the human bottleneck for contact tracing
Source: PNAS Nexus. 2024 Jul 16;3(7):pgae283. doi: 10.1093/pnasnexus/pgae283 (PMC11285183; doi:10.1093/pnasnexus/pgae283)
Supplement: pgae283_Supplementary_Data [file pgae283_supplementary_data.pdf]

Supporting Information

Extended Methods

*Subjects***.** Data was collected anonymously, in adherence to the Declaration of Helsinki and only from subjects who provided informed consent. Subjects were compensated by the panel provider (see below) and the local institutional review board has approved the study (lokale Ethik-Kommission des Fachbereichs 06 der Justus Liebig Universität Gießen (JLU) 2020-0029).

Panel subjects were recruited in collaboration with ZPID PsychLab online (https://leibniz-psychology.org/en/services/data-collection/psychlab-online/), which purchased the sample from commercial panel provider respondi (https://www.respondi.com/EN/). Respondi recruited and incentivised subjects from their quality controlled online panel and directed them to the online CTIs programmed by the authors and independently hosted at SoSciSurvey (https://www.soscisurvey.de). The characteristics of the panel adhere to standards for online access panels determined by ISO 20252:2009 or ISO 20252:2019. These standards require access to full disclosure of incentive terms and conditions applying to the project in the invitation email that contains the link to the survey. Incentives are provided in the form of tokens or bonus points that, after a certain amount has been accumulated, can be monetized, transformed into a voucher or donated (choice of the participant).

The panel provider aimed at recruiting age and gender representative samples of the adult populations of the UK and Germany aged 18-74, with a size of n = 7,500 each. Pre-registered power simulations (https://doi.org/10.23668/psycharchives.12658) suggested that this sample size allows accurate recovery of modest memory decline, even if the day-to-day fluctuation of individual contacts are orders of magnitude higher than the memory effects (as was suggested by pilot data).

Only subjects who passed an initial attention check proceeded to the online CTI. We recorded a total of 37,282 visits to the landing page of the survey. 28,247 visitors passed the initial attention check and provided demographic information, 19,456 proceeded to give informed consent to participate in the study (only possible if the respective quotas were not filled already, see below) and 15,015 subjects went on to complete the questionnaire. 13,407 of those were included in the final analysis. The remaining 1,608 subjects who completed the survey were excluded because at least one of the following criteria applied to them: They reported negative contact numbers; they reported a total of zero contacts; they indicated that they did not fill out the questionnaire diligently in an exit question; they reported a total number of contacts that was at least 7.5 median absolute deviations higher than the corresponding median of their sample (i.e. a robustly estimated z-value of at least 5). The included UK sample comprised 6,733 subjects (3,425 females) from 18-74 years with a median age of 45. The included GER sample comprised 6,674 subjects (3,373 females) from 18-74 years with a median age of 47.

The age- and gender-quotas the panel provider aimed for and those in the included sample were as follows:

Germany: female 49.79% (included 49.46%), 18-29 years 18.45% (18.61%), 30-39 years 18.12% (18.10%), 40-49 years 16.78% (15.27%), 50-59 years 22.17% (20.00%), 60-74 years 24.47% (24.49%).

UK: female 50.40% (included 49.13%), 18-39 years 40.72% (39.30%; note that the panel provider did not offer a narrower recruitment quota for the youngest UK sample; included participants in this bracket broke down into 12.09% 18-29-year-olds and 27.21% 30-39-year-olds), 40-49 years 18.31% (15.97%), 50-59 years 19.05% (17.04%), 60-74 years 21.93% (23.07%).

Note that attrition due to the attention check, necessity for informed consent and other exclusion criteria likely biased our sample towards motivated subjects. This underscores the caveat that our estimates should be interpreted as a lower bound of underreporting.

*Survey***.** The online survey was designed in consultation with local health officials (Gesundheitsamt, Landkreis Giessen, Germany) to emulate a traditional Contact Tracing Interview (CTI). Specifically, subjects who provide informed consent filled in a questionnaire providing a definition of relevant contacts and asking about their number for each of the 14 preceding days, separately for increasingly wider domains of potential social interaction.

The survey first asked subjects to make use of memory aids where possible and to note relevant contacts on a sheet of paper, before counting their number and entering them into the survey. It then provided three criteria, at least one of which needed to be fulfilled for a contact to be relevant: a person with whom the participant had an in-person conversation at a distance of less than 1.5m, spent at least 10 minutes with at a distance of less than 1.5 m, or spent more than 10 minutes with in a closed room. Subjects were further instructed to only report contacts they could specifically remember and name. Due to an editing error, only the German survey mentioned the ability to name contacts as a requirement, which may explain the somewhat higher intercept of reported contacts in the UK sample. Reassuringly, all patterns of reporting decline replicated between the two samples and thus appear robust to this difference. Subjects were also informed that they should count a given contact only under the first applicable domain for a given day (for instance, a household member who is also a family member should only be counted as household member).

Subjects were provided with separate questionnaire pages for each of the preceding 14 days, asking about the number of remembered relevant contacts, separately for those among members of the household, family and friends, contacts at work or school, medical settings, public transport, shopping, leisure and other types of contacts. Subjects were also asked to indicate the corresponding day of the week. Once subjects provided information on all of the preceding 14 days, they were asked to rate for how many days they made use of memory aids such as a scheduler on a 5-point scale (No days; Less than half; About half; More than half; All days). Finally, they were asked to indicate whether they completed the questionnaire diligently.

Please see the Appendix below for a copy of the English and German versions of the survey.

Preregistration. We preregistered the design of our study, an analysis plan and power analyses based on pilot data (https://doi.org/10.23668/psycharchives.12658). Several aspects of our analysis deviate from and extend the preregistered plan. Most importantly, we added aid use as a moderator and harnessed the power afforded by our large samples with Generalised Linear Mixed Effects models (in addition to polynomial fits to the daily averages of reported contacts).

Appendix A: Survey – English version

JUSTUS-LIEBIG-UNIVERSITÄT GIESSEN

0% completed

Dear participants,
Thank you for taking the time to participate in our study!

Before you can start, we kindly ask you to solve two short puzzles. This is solely to identify you as a human being.
Please understand that you can only participate in the study if you solve both puzzles correctly.

0	0	0	0	1
0	0	1	0	1
1	1	1	0	1
0	1	1	1	0
0	0	1	1	1

How many zeros are in this table?

Please note that after 3 incorrect answers, participation in this questionnaire will no longer be possible.

Next

JUSTUS-LIEBIG-UNIVERSITÄT GIESSEN

Alright, let's get started with the questionnaire after the next puzzle...

1	0	1	0	1
1	0	1	0	0
1	1	0	1	0
0	0	1	0	1
1	1	0	0	0

How many zeros are in this table?

Please note that after 3 incorrect answers, participation in this questionnaire will no longer be possible.

Back

Next

JUSTUS-LIEBIG-
UNIVERSITÄT
GIESSEN

8% completed

Before you can start the questionnaire, we still need some information about you.

What is your sex?

- ◯ female
- ◯ male
- ◯ diverse

How old are you?

I am [ ] years old

Back

Next

JUSTUS-LIEBIG-UNIVERSITÄT GIESSEN

13% completed

Dear participants,

Thank you for taking the time to participate in our study!

We aim to investigate how well people can remember their contacts from the last 14 days. This question has been scarcely explored and can be crucial in the context of contagious diseases such as COVID-19. **Please read the following instructions carefully and answer the questions as accurately as possible. By doing so, you are making an important contribution to the fight against contagious diseases.** A tip: If you use a planner or something similar, you can use it as a memory aid. Please have paper and pen ready to complete the questionnaire. Write down your contacts and then enter the number in the questionnaire. Please use any tool that is available to you!

First, we will ask you about the number of your relevant contacts for each of the last 14 days. Relevant contacts are those that meet **at least one of the following criteria:**

People

- with whom you had a **conversation** (distance < 1.5m)

- with whom you had **close contact for more than 10 minutes** (distance < 1.5m)

- with whom you spent **more than 10 minutes in a closed room**

Very important: **Please only list contacts that you can specifically remember** (for example, a co-worker sitting next to you). For example, if you were on a bus and don't remember if your travel time overlapped with other passengers for more than 10 minutes, please count it as '0' relevant contacts.

We will ask for your contacts separately for several areas. If a contact falls under multiple categories, please list them only **once** under the first applicable category. For example, a household and family member should only be counted under household members.

Very important: We will never ask you to identify yourself or your contacts. Our questionnaire is strictly anonymous!

Back

Next

JUSTUS-LIEBIG-UNIVERSITÄT GIESSEN

17% completed

Consent form

Please read and print or save the following consent form before proceeding to the questionnaire:
consent form

Responsible for study

If you have any questions, please feel free to contact one of the following persons:
Maximilian Broda, Experimental Psychology, Justus Liebig University Giessen, Maximilian.Broda@psychol.uni-giessen.de
oder
Benjamin de Haas, Experimental Psychology, Justus Liebig University Giessen, Benjamin.de-Haas@psychol.uni-giessen.de

If you agree with the participation conditions described in the consent form, please confirm the following options to participate in this study. If you do not agree, please close the current browser window to abort the study.

- [ ] I confirm that I have received, carefully read and understood information regarding the nature and aim of the study, including participant requirements and possible adverse effects. I had sufficient opportunity to ask additional questions and received understandable answers from the responsible researcher (see above for contact details). I received a copy of the study information. I had sufficient time to decide for or against taking part in the study. By clicking this checkbox I confirm that I understood the study intent and information and that I agree to participate voluntarily. I understand that I can abort my participation at any point without having to provide a reason and without personal disadvantage. The responsible researcher can equally abort the task at any point. I understood that the primary aim of this study is basic research and that it may not cause personal benefits for me.
- [ ] By clicking this checkbox I agree to the storage, processing and publication of the anonymous data I provide.
- [ ] By clicking this checkbox I confirm that I am at least 18 years old and am proficient in English.

Back

Next

Please carefully go through each contact category of a given day and try to remember as many relevant contacts as possible. Then enter the respective number of relevant contacts.

Relevant contacts are those that meet at least one of the following criteria:

People

- with whom you had a **conversation** (distance < 1.5m)

- with whom you had **close contact for more than 10 minutes** (distance < 1.5m)

- with whom you spent **more than 10 minutes in a closed room**

Please only list contacts that you specifically remember.

Back Next

JUSTUS-LIEBIG-
UNIVERSITÄT
GIESSEN

26% completed

Please enter the number of relevant contacts from **yesterday** that you specifically remember.

What day of the week was it?

[Please choose]

Relevant contacts are those that meet **at least one of the following criteria**:

People
-with whom you had a **conversation** (distance < 1.5m)
-with whom you had **close contact for more than 10 minutes** (distance < 1.5m)
-with whom you spent **more than 10 minutes in a closed room**

If you did not have any contacts in a category, please enter **0**.

Here you can review the categories again if you are unsure.

	Number of remembered and known contacts
household members	
family / friends	
contacts at work or school	
medical settings (e.g. doctor's appointment)	
public transport	
shopping	
leisure (e.g. sports, movie theater visit)	
other types of contacts	

Back

Next

JUSTUS-LIEBIG-
UNIVERSITÄT
GIESSEN

30% completed

Please enter the number of relevant contacts from **two days ago** that you specifically remember.

What day of the week was it?

[Please choose]

Relevant contacts are those that meet **at least one of the following criteria**:

People
-with whom you had a **conversation** (distance < 1.5m)
-with whom you had **close contact for more than 10 minutes** (distance < 1.5m)
-with whom you spent **more than 10 minutes in a closed room**

If you did not have any contacts in a category, please enter **0**.

Here you can review the categories again if you are unsure.

	Number of remembered and known contacts
household members	
family / friends	
contacts at work or school	
medical settings (e.g. doctor's appointment)	
public transport	
shopping	
leisure (e.g. sports, movie theater visit)	
other types of contacts	

Back

Next

JUSTUS-LIEBIG-
UNIVERSITÄT
GIESSEN

34% completed

Please enter the number of relevant contacts from **three days ago** that you specifically remember.

What day of the week was it?

[Please choose]

Relevant contacts are those that meet **at least one of the following criteria**:

People
-with whom you had a **conversation** (distance < 1.5m)
-with whom you had **close contact for more than 10 minutes** (distance < 1.5m)
-with whom you spent **more than 10 minutes in a closed room**

If you did not have any contacts in a category, please enter **0**.

Here you can review the categories again if you are unsure.

	Number of remembered and known contacts
household members	
family / friends	
contacts at work or school	
medical settings (e.g. doctor's appointment)	
public transport	
shopping	
leisure (e.g. sports, movie theater visit)	
other types of contacts	

Back Next

JUSTUS-LIEBIG-UNIVERSITÄT GIESSEN

39% completed

Please enter the number of relevant contacts from **four days ago** that you specifically remember.

What day of the week was it?

[Please choose]

Relevant contacts are those that meet **at least one of the following criteria**:

People
-with whom you had a **conversation** (distance < 1.5m)
-with whom you had **close contact for more than 10 minutes** (distance < 1.5m)
-with whom you spent **more than 10 minutes in a closed room**

If you did not have any contacts in a category, please enter **0**.

Here you can review the categories again if you are unsure.

	Number of remembered and known contacts
household members	
family / friends	
contacts at work or school	
medical settings (e.g. doctor's appointment)	
public transport	
shopping	
leisure (e.g. sports, movie theater visit)	
other types of contacts	

Back

Next

JUSTUS-LIEBIG-
UNIVERSITÄT
GIESSEN

43% completed

Please enter the number of relevant contacts from **five days ago** that you specifically remember.

What day of the week was it?

[Please choose]

Relevant contacts are those that meet **at least one of the following criteria**:

People
-with whom you had a **conversation** (distance < 1.5m)
-with whom you had **close contact for more than 10 minutes** (distance < 1.5m)
-with whom you spent **more than 10 minutes in a closed room**

If you did not have any contacts in a category, please enter **0**.

Here you can review the categories again if you are unsure.

	Number of remembered and known contacts
household members	
family / friends	
contacts at work or school	
medical settings (e.g. doctor’s appointment)	
public transport	
shopping	
leisure (e.g. sports, movie theater visit)	
other types of contacts	

Back

Next

JUSTUS-LIEBIG-UNIVERSITÄT GIESSEN

48% completed

Please enter the number of relevant contacts from **six days ago** that you specifically remember.

What day of the week was it?

[Please choose]

Relevant contacts are those that meet **at least one of the following criteria**:

People
-with whom you had a **conversation** (distance < 1.5m)
-with whom you had **close contact for more than 10 minutes** (distance < 1.5m)
-with whom you spent **more than 10 minutes in a closed room**

If you did not have any contacts in a category, please enter **0**.

Here you can review the categories again if you are unsure.

	Number of remembered and known contacts
household members	
family / friends	
contacts at work or school	
medical settings (e.g. doctor's appointment)	
public transport	
shopping	
leisure (e.g. sports, movie theater visit)	
other types of contacts	

Back Next

JUSTUS-LIEBIG-UNIVERSITÄT GIESSEN

52% completed

Please enter the number of relevant contacts from **seven days ago** that you specifically remember.

What day of the week was it?

[Please choose]

Relevant contacts are those that meet **at least one of the following criteria**:

People
-with whom you had a **conversation** (distance < 1.5m)
-with whom you had **close contact for more than 10 minutes** (distance < 1.5m)
-with whom you spent **more than 10 minutes in a closed room**

If you did not have any contacts in a category, please enter **0**.

Here you can review the categories again if you are unsure.

	Number of remembered and known contacts
household members	
family / friends	
contacts at work or school	
medical settings (e.g. doctor's appointment)	
public transport	
shopping	
leisure (e.g. sports, movie theater visit)	
other types of contacts	

Back

Next

JUSTUS-LIEBIG-
UNIVERSITÄT
GIESSEN

56% completed

Please enter the number of relevant contacts from **eight days ago** that you specifically remember.

What day of the week was it?

[Please choose]

Relevant contacts are those that meet **at least one of the following criteria**:

People
-with whom you had a **conversation** (distance < 1.5m)
-with whom you had **close contact for more than 10 minutes** (distance < 1.5m)
-with whom you spent **more than 10 minutes in a closed room**

If you did not have any contacts in a category, please enter **0**.

Here you can review the categories again if you are unsure.

	Number of remembered and known contacts
household members	
family / friends	
contacts at work or school	
medical settings (e.g. doctor's appointment)	
public transport	
shopping	
leisure (e.g. sports, movie theater visit)	
other types of contacts	

Back

Next

JUSTUS-LIEBIG-UNIVERSITÄT GIESSEN

61% completed

Please enter the number of relevant contacts from **nine days ago** that you specifically remember.

What day of the week was it?

[Please choose]

Relevant contacts are those that meet **at least one of the following criteria**:

People
-with whom you had a **conversation** (distance < 1.5m)
-with whom you had **close contact for more than 10 minutes** (distance < 1.5m)
-with whom you spent **more than 10 minutes in a closed room**

If you did not have any contacts in a category, please enter **0**.

Here you can review the categories again if you are unsure.

	Number of remembered and known contacts
household members	
family / friends	
contacts at work or school	
medical settings (e.g. doctor's appointment)	
public transport	
shopping	
leisure (e.g. sports, movie theater visit)	
other types of contacts	

Back

Next

JUSTUS-LIEBIG-UNIVERSITÄT GIESSEN

65% completed

Please enter the number of relevant contacts from **ten days ago** that you specifically remember.

What day of the week was it?

[Please choose]

Relevant contacts are those that meet **at least one of the following criteria**:

People
-with whom you had a **conversation** (distance < 1.5m)
-with whom you had **close contact for more than 10 minutes** (distance < 1.5m)
-with whom you spent **more than 10 minutes in a closed room**

If you did not have any contacts in a category, please enter **0**.

Here you can review the categories again if you are unsure.

	Number of remembered and known contacts
household members	
family / friends	
contacts at work or school	
medical settings (e.g. doctor's appointment)	
public transport	
shopping	
leisure (e.g. sports, movie theater visit)	
other types of contacts	

Back Next

Please enter the number of relevant contacts from **eleven days ago** that you specifically remember.

What day of the week was it?

[Please choose]

Relevant contacts are those that meet **at least one of the following criteria**:

People
-with whom you had a **conversation** (distance < 1.5m)
-with whom you had **close contact for more than 10 minutes** (distance < 1.5m)
-with whom you spent **more than 10 minutes in a closed room**

If you did not have any contacts in a category, please enter **0**.

Here you can review the categories again if you are unsure.

	Number of remembered and known contacts
household members	
family / friends	
contacts at work or school	
medical settings (e.g. doctor's appointment)	
public transport	
shopping	
leisure (e.g. sports, movie theater visit)	
other types of contacts	

Back

Next

JUSTUS-LIEBIG-UNIVERSITÄT GIESSEN

74% completed

Please enter the number of relevant contacts from **twelve days ago** that you specifically remember.

What day of the week was it?

[Please choose]

Relevant contacts are those that meet **at least one of the following criteria**:

People
-with whom you had a **conversation** (distance < 1.5m)
-with whom you had **close contact for more than 10 minutes** (distance < 1.5m)
-with whom you spent **more than 10 minutes in a closed room**

If you did not have any contacts in a category, please enter **0**.

Here you can review the categories again if you are unsure.

	Number of remembered and known contacts
household members	
family / friends	
contacts at work or school	
medical settings (e.g. doctor's appointment)	
public transport	
shopping	
leisure (e.g. sports, movie theater visit)	
other types of contacts	

Back

Next

JUSTUS-LIEBIG-UNIVERSITÄT GIESSEN

78% completed

Please enter the number of relevant contacts from **13 days ago** that you specifically remember.

What day of the week was it?

[Please choose]

Relevant contacts are those that meet **at least one of the following criteria**:

People
-with whom you had a **conversation** (distance < 1.5m)
-with whom you had **close contact for more than 10 minutes** (distance < 1.5m)
-with whom you spent **more than 10 minutes in a closed room**

If you did not have any contacts in a category, please enter **0**.

Here you can review the categories again if you are unsure.

	Number of remembered and known contacts
household members	
family / friends	
contacts at work or school	
medical settings (e.g. doctor's appointment)	
public transport	
shopping	
leisure (e.g. sports, movie theater visit)	
other types of contacts	

Back Next

JUSTUS-LIEBIG-
UNIVERSITÄT
GIESSEN

83% completed

Please enter the number of relevant contacts from **14 days ago** that you specifically remember.

What day of the week was it?

[Please choose]

Relevant contacts are those that meet **at least one of the following criteria**:

People
-with whom you had a **conversation** (distance < 1.5m)
-with whom you had **close contact for more than 10 minutes** (distance < 1.5m)
-with whom you spent **more than 10 minutes in a closed room**

If you did not have any contacts in a category, please enter **0**.

Here you can review the categories again if you are unsure.

	Number of remembered and known contacts
household members	
family / friends	
contacts at work or school	
medical settings (e.g. doctor's appointment)	
public transport	
shopping	
leisure (e.g. sports, movie theater visit)	
other types of contacts	

Back

Next

JUSTUS-LIEBIG-
UNIVERSITÄT
GIESSEN

87% completed

For how many days did you use aids (e.g. calendar) to determine your contacts?

[Please choose]

Back

Next

JUSTUS-LIEBIG-
UNIVERSITÄT
GIESSEN

Thank you very much for completing our questionnaire!

For our research, it is very important to know whether you filled in this questionnaire diligently. You will receive your incentive regardless of how you answer the following question, so please respond honestly. **This information will not be shared with respondi.**

I have completed the questionnaire diligently

○ yes ○ no

Back Next

Appendix B: Survey – German version

JUSTUS-LIEBIG-UNIVERSITÄT GIESSEN

4% ausgefüllt

Sehr geehrte Teilnehmende,

Vielen Dank, dass Sie sich Zeit für unsere Studie nehmen!

Bevor Sie beginnen können, möchten wir Sie bitten zwei kurze Rätsel zu lösen. Dies dient einzig dazu, Sie als Mensch zu identifizieren. Bitte haben Sie Verständnis dafür, dass Sie nur an der Studie teilnehmen können, wenn Sie beide Rätsel korrekt lösen.

0	0	0	0	1
0	0	1	0	1
1	1	1	0	1
0	1	1	1	0
0	0	1	1	1

Wie viele „0“ sehen Sie?

Bitte beachten Sie, dass nach 3 falschen Antworten eine Teilnahme an diesem Fragebogen nicht mehr möglich ist.

Weiter

Gleich kann es mit dem Fragebogen losgehen...

1	0	1	0	1
1	0	1	0	0
1	1	0	1	0
0	0	1	0	1
1	1	0	0	0

Wie viele „0“ sehen Sie hier?

Bitte beachten Sie, dass nach 3 falschen Antworten eine Teilnahme an diesem Fragebogen nicht mehr möglich ist.

Zurück

Weiter

Sehr geehrte Teilnehmende,

Vielen Dank, dass Sie sich Zeit für unsere Studie nehmen!

Wir möchten herausfinden, wie gut Menschen Ihre Kontakte der letzten 14 Tage erinnern können. Diese Frage ist bisher kaum untersucht und kann im Zusammenhang ansteckender Krankheiten wie CoViD-19 entscheidend sein. **Bitte lesen Sie die folgenden Instruktionen aufmerksam und beantworten die Fragen so genau wie möglich. Sie leisten damit einen wichtigen Beitrag zur Bekämpfung ansteckender Krankheiten.** Ein Tipp: Falls Sie einen Terminplaner oder ähnliches nutzen, können Sie ihn als Gedächtnisstütze nutzen. Bitte haben Sie für die Bearbeitung des Fragebogens Zettel und Stift zur Hand. Notieren Sie zunächst Ihre Kontakte und tragen dann die Anzahl in den Fragebogen ein. Bitte machen Sie Gebrauch von jedem Hilfsmittel, das Ihnen zur Verfügung steht!

Im Folgenden werden wir nach der Zahl Ihrer relevanten Kontakte für jeden der letzten 14 Tage fragen. Relevant sind nur Kontakte auf die **mindestens eins der folgenden Kriterien zutrifft**:
Menschen

- mit denen Sie ein **Gespräch** geführt haben (Abstand < 1,5m)
- mit denen Sie **mehr als 10 Minuten engen Kontakt** hatten (Abstand < 1,5m)
- mit denen Sie **mehr als 10 Minuten in einem geschlossenen Raum** verbracht haben

Bitte geben Sie ausschließlich Kontakte an, die Sie benennen können (beispielsweise eine Sitznachbarin im Büro). Ganz wichtig: **Bitte geben Sie nur Kontakte an, die Sie konkret und sicher erinnern können.** Wenn Sie zum Beispiel im Bus waren und nicht mehr genau wissen, ob sich Ihre Fahrzeit mehr als 10 Minuten mit der anderer Passagiere überschnitten hat, zählen Sie dies bitte als '0' relevante Kontakte.

Wir fragen Ihre Kontakte getrennt nach mehreren Bereichen ab. Fällt ein Kontakt unter mehrere Kategorien, geben Sie ihn bitte nur **einmal**, unter der jeweils ersten Kategorie an. Beispiel: Ein Haushalts- und Familienmitglied wird nur unter Haushaltsmitgliedern gezählt.

Zuletzt bitten wir Sie für jeden Tag anzugeben, ob es sich um einen gesetzlichen Feiertag / Wochenende handelte.

Ganz wichtig: Wir werden Sie niemals bitten sich selbst oder Ihre Kontakte zu identifizieren. Unser Fragebogen ist streng anonym!

Zurück Weiter

JUSTUS-LIEBIG-UNIVERSITÄT GIESSEN

16% ausgefüllt

Einwilligungserklärung

Bitte lesen und drucken oder speichern Sie folgende Einwilligungserklärung, bevor Sie mit dem Fragebogen fortfahren:
Einwilligungserklärung

Studienleitung
Bei Rückfragen wenden Sie sich gerne an die Studienleitung:
Maximilian Broda, Allgemeine Psychologie I, Justus-Liebig-Universität Gießen, Maximilian.Broda@psychol.uni-giessen.de
oder
Benjamin de Haas, Allgemeine Psychologie I, Justus-Liebig-Universität Gießen, Benjamin.de-Haas@psychol.uni-giessen.de

Wenn Sie mit den in der Einwilligungserklärung beschriebenen Bedingungen der Teilnahme einverstanden sind, bestätigen Sie bitte folgende Optionen, um an dieser Studie teilzunehmen. Wenn Sie nicht einverstanden sind, schließen Sie bitte das aktuelle Browserfenster, um die Studie abzubrechen.

- [ ] Ich habe die Studieninformation über Ziel und Ablauf der Untersuchung sowie studienbedingte Erfordernisse und mögliche Nebenwirkungen erhalten, gründlich durchgelesen und verstanden. Ich hatte ausreichend Gelegenheit, mich bei dem Versuchsleiter über den Untersuchungshergang zu informieren, sowie auftretende Fragen zu stellen (s.o. für Kontaktinformationen). Diese wurden mir von dem Versuchsleiter verständlich beantwortet. Eine Kopie der Probandeninformation habe ich erhalten. Ich hatte ausreichend Zeit, mich für oder gegen eine Teilnahme an dieser Studie zu entscheiden. Hiermit erkläre ich, dass ich das Vorhaben und die Information verstanden habe und freiwillig an der Studie teilnehme. Ich habe verstanden, dass ich jederzeit ohne Angabe von Gründen aus der Studie ausscheiden kann, ohne dass mir persönliche Nachteile entstehen. Auch der Versuchsleiter kann die Studie jederzeit beenden. Mir ist bekannt, dass diese Studie in erster Linie der Wissenserweiterung dient und gegebenenfalls auch keinen persönlichen Vorteil für mich bringen kann.
- [ ] Hiermit willige ich in die Speicherung, Verarbeitung und Veröffentlichung meiner anonymisierten Daten ein.
- [ ] Hiermit bestätige ich, dass ich mindestens 18 Jahre alt bin und über gute Deutschkenntnisse verfüge.

Zurück

Weiter

JUSTUS-LIEBIG-UNIVERSITÄT GIESSEN

20% ausgefüllt

Bevor Sie mit dem Fragebogen starten können, benötigen wir noch ein paar Informationen über Ihre Person.

Welches Geschlecht haben Sie?

- ○ weiblich
- ○ männlich
- ○ divers

Wie alt sind Sie?

Ich bin [ ] Jahre alt

Zurück

Weiter

JUSTUS-LIEBIG-UNIVERSITÄT GIESSEN

24% ausgefüllt

Bitte gehen Sie nun jede Kontaktkategorie eines Tages sorgfältig durch und versuchen so viele relevante Kontakte wie möglich zu erinnern. Dann tragen Sie die jeweilige Anzahl der relevanten Kontakte ein.

Relevant sind nur Kontakte auf die mindestens eins der folgenden Kriterien zutrifft:

Menschen
- mit denen Sie ein **Gespräch** geführt haben (Abstand < 1,5m)
- mit denen Sie **mehr als 10 Minuten engen Kontakt** hatten (Abstand < 1,5m)
- mit denen Sie **mehr als 10 Minuten in einem geschlossenen Raum** verbracht haben

Bitte zählen Sie nur relevante Kontakte, die Sie tatsächlich sicher erinnern können.

Zurück

Weiter

JUSTUS-LIEBIG-UNIVERSITÄT GIESSEN

29% ausgefüllt

Bitte geben Sie nun die Anzahl relevanter Kontakte **von gestern** an, an die Sie sich konkret erinnern können.

Um welchen Wochentag handelte es sich?

[Bitte auswählen]

Relevant sind nur Kontakte auf die **mindestens eins der folgenden Kriterien zutrifft**:

Menschen
- mit denen Sie ein **Gespräch** geführt haben (Abstand < 1,5m)
- mit denen Sie **mehr als 10 Minuten engen Kontakt** hatten (Abstand < 1,5m)
- mit denen Sie **mehr als 10 Minuten in einem geschlossenen Raum** verbracht haben

Sollten Sie keine Kontakte einer Kategorie gehabt haben, tragen Sie bitte eine **0** ein.

Hier können Sie sich die Kategorien erneut ansehen, wenn Sie unsicher sind.

	Anzahl erinnerter und namentlich bekannter Kontaktpersonen
Haushaltsmitglied	
Familie / Freunde	
Arbeits- / Schulkontakte	
Medizinische Versorgung (z.B. Arztbesuch)	
Öffentliche Verkehrsmittel	
Einkaufen	
Freizeitaktivitäten (z.B. Sport, Kinobesuch)	
Andere	

Zurück

Weiter

JUSTUS-LIEBIG-UNIVERSITÄT GIESSEN

33% ausgefüllt

Bitte geben Sie nun die Anzahl relevanter Kontakte **vor zwei Tagen** an, an die Sie sich konkret erinnern können.

Um welchen Wochentag handelte es sich?

[Bitte auswählen]

Relevant sind nur Kontakte auf die **mindestens eins der folgenden Kriterien zutrifft**:

Menschen
- mit denen Sie ein **Gespräch** geführt haben (Abstand < 1,5m)
- mit denen Sie **mehr als 10 Minuten engen Kontakt** hatten (Abstand < 1,5m)
- mit denen Sie **mehr als 10 Minuten in einem geschlossenen Raum** verbracht haben

Sollten Sie keine Kontakte einer Kategorie gehabt haben, tragen Sie bitte eine **0** ein.

Hier können Sie sich die Kategorien erneut ansehen, wenn Sie unsicher sind.

	Anzahl erinnerter und namentlich bekannter Kontaktpersonen
Haushaltsmitglied	
Familie / Freunde	
Arbeits- / Schulkontakte	
Medizinische Versorgung (z.B. Arztbesuch)	
Öffentliche Verkehrsmittel	
Einkaufen	
Freizeitaktivitäten (z.B. Sport, Kinobesuch)	
Andere	

Zurück Weiter

JUSTUS-LIEBIG-UNIVERSITÄT GIESSEN

37% ausgefüllt

Bitte geben Sie nun die Anzahl relevanter Kontakte **vor drei Tagen** an, an die Sie sich konkret erinnern können.

Um welchen Wochentag handelte es sich?

[Bitte auswählen]

Relevant sind nur Kontakte auf die **mindestens eins der folgenden Kriterien zutrifft**:

Menschen
- mit denen Sie ein **Gespräch** geführt haben (Abstand < 1,5m)
- mit denen Sie **mehr als 10 Minuten engen Kontakt** hatten (Abstand < 1,5m)
- mit denen Sie **mehr als 10 Minuten in einem geschlossenen Raum** verbracht haben

Sollten Sie keine Kontakte einer Kategorie gehabt haben, tragen Sie bitte eine **0** ein.

Hier können Sie sich die Kategorien erneut ansehen, wenn Sie unsicher sind.

	Anzahl erinnerter und namentlich bekannter Kontaktpersonen
Haushaltsmitglied	
Familie / Freunde	
Arbeits- / Schulkontakte	
Medizinische Versorgung (z.B. Arztbesuch)	
Öffentliche Verkehrsmittel	
Einkaufen	
Freizeitaktivitäten (z.B. Sport, Kinobesuch)	
Andere	

Zurück

Weiter

JUSTUS-LIEBIG-UNIVERSITÄT GIESSEN

41% ausgefüllt

Bitte geben Sie nun die Anzahl relevanter Kontakte **vor vier Tagen** an, an die Sie sich konkret erinnern können.

Um welchen Wochentag handelte es sich?

[Bitte auswählen]

Relevant sind nur Kontakte auf die **mindestens eins der folgenden Kriterien zutrifft**:

Menschen
- mit denen Sie ein **Gespräch** geführt haben (Abstand < 1,5m)
- mit denen Sie **mehr als 10 Minuten engen Kontakt** hatten (Abstand < 1,5m)
- mit denen Sie **mehr als 10 Minuten in einem geschlossenen Raum** verbracht haben

Sollten Sie keine Kontakte einer Kategorie gehabt haben, tragen Sie bitte eine **0** ein.

Hier können Sie sich die Kategorien erneut ansehen, wenn Sie unsicher sind.

	Anzahl erinnerter und namentlich bekannter Kontaktpersonen
Haushaltsmitglied	
Familie / Freunde	
Arbeits- / Schulkontakte	
Medizinische Versorgung (z.B. Arztbesuch)	
Öffentliche Verkehrsmittel	
Einkaufen	
Freizeitaktivitäten (z.B. Sport, Kinobesuch)	
Andere	

Zurück Weiter

JUSTUS-LIEBIG-
UNIVERSITÄT
GIESSEN

45% ausgefüllt

Bitte geben Sie nun die Anzahl relevanter Kontakte **vor fünf Tagen** an, an die Sie sich konkret erinnern können.

Um welchen Wochentag handelte es sich?

[Bitte auswählen]

Relevant sind nur Kontakte auf die **mindestens eins der folgenden Kriterien zutrifft**:

Menschen
- mit denen Sie ein **Gespräch** geführt haben (Abstand < 1,5m)
- mit denen Sie **mehr als 10 Minuten engen Kontakt** hatten (Abstand < 1,5m)
- mit denen Sie **mehr als 10 Minuten in einem geschlossenen Raum** verbracht haben

Sollten Sie keine Kontakte einer Kategorie gehabt haben, tragen Sie bitte eine **0** ein.

Hier können Sie sich die Kategorien erneut ansehen, wenn Sie unsicher sind.

	Anzahl erinnerter und namentlich bekannter Kontaktpersonen
Haushaltsmitglied	
Familie / Freunde	
Arbeits- / Schulkontakte	
Medizinische Versorgung (z.B. Arztbesuch)	
Öffentliche Verkehrsmittel	
Einkaufen	
Freizeitaktivitäten (z.B. Sport, Kinobesuch)	
Andere	

Zurück

Weiter

JUSTUS-LIEBIG-UNIVERSITÄT GIESSEN

50% ausgefüllt

Bitte geben Sie nun die Anzahl relevanter Kontakte **vor sechs Tagen** an, an die Sie sich konkret erinnern können.

Um welchen Wochentag handelte es sich?

[Bitte auswählen]

Relevant sind nur Kontakte auf die **mindestens eins der folgenden Kriterien zutrifft**:

Menschen
- mit denen Sie ein **Gespräch** geführt haben (Abstand < 1,5m)
- mit denen Sie **mehr als 10 Minuten engen Kontakt** hatten (Abstand < 1,5m)
- mit denen Sie **mehr als 10 Minuten in einem geschlossenen Raum** verbracht haben

Sollten Sie keine Kontakte einer Kategorie gehabt haben, tragen Sie bitte eine **0** ein.

Hier können Sie sich die Kategorien erneut ansehen, wenn Sie unsicher sind.

	Anzahl erinnerter und namentlich bekannter Kontaktpersonen
Haushaltsmitglied	
Familie / Freunde	
Arbeits- / Schulkontakte	
Medizinische Versorgung (z.B. Arztbesuch)	
Öffentliche Verkehrsmittel	
Einkaufen	
Freizeitaktivitäten (z.B. Sport, Kinobesuch)	
Andere	

Zurück

Weiter

JUSTUS-LIEBIG-UNIVERSITÄT GIESSEN

54% ausgefüllt

Bitte geben Sie nun die Anzahl relevanter Kontakte **vor sieben Tagen** an, an die Sie sich konkret erinnern können.

Um welchen Wochentag handelte es sich?

[Bitte auswählen]

Relevant sind nur Kontakte auf die **mindestens eins der folgenden Kriterien zutrifft**:

Menschen
- mit denen Sie ein **Gespräch** geführt haben (Abstand < 1,5m)
- mit denen Sie **mehr als 10 Minuten engen Kontakt** hatten (Abstand < 1,5m)
- mit denen Sie **mehr als 10 Minuten in einem geschlossenen Raum** verbracht haben

Sollten Sie keine Kontakte einer Kategorie gehabt haben, tragen Sie bitte eine **0** ein.

Hier können Sie sich die Kategorien erneut ansehen, wenn Sie unsicher sind.

	Anzahl erinnerter und namentlich bekannter Kontaktpersonen
Haushaltsmitglied	
Familie / Freunde	
Arbeits- / Schulkontakte	
Medizinische Versorgung (z.B. Arztbesuch)	
Öffentliche Verkehrsmittel	
Einkaufen	
Freizeitaktivitäten (z.B. Sport, Kinobesuch)	
Andere	

Zurück   Weiter

JUSTUS-LIEBIG-UNIVERSITÄT GIESSEN

58% ausgefüllt

Bitte geben Sie nun die Anzahl relevanter Kontakte **vor acht Tagen** an, an die Sie sich konkret erinnern können.

Um welchen Wochentag handelte es sich?

[Bitte auswählen]

Relevant sind nur Kontakte auf die **mindestens eins der folgenden Kriterien zutrifft**:

Menschen
- mit denen Sie ein **Gespräch** geführt haben (Abstand < 1,5m)
- mit denen Sie **mehr als 10 Minuten engen Kontakt** hatten (Abstand < 1,5m)
- mit denen Sie **mehr als 10 Minuten in einem geschlossenen Raum** verbracht haben

Sollten Sie keine Kontakte einer Kategorie gehabt haben, tragen Sie bitte eine **0** ein.

Hier können Sie sich die Kategorien erneut ansehen, wenn Sie unsicher sind.

	Anzahl erinnerter und namentlich bekannter Kontaktpersonen
Haushaltsmitglied	
Familie / Freunde	
Arbeits- / Schulkontakte	
Medizinische Versorgung (z.B. Arztbesuch)	
Öffentliche Verkehrsmittel	
Einkaufen	
Freizeitaktivitäten (z.B. Sport, Kinobesuch)	
Andere	

Zurück Weiter

JUSTUS-LIEBIG-
UNIVERSITÄT
GIESSEN

62% ausgefüllt

Bitte geben Sie nun die Anzahl relevanter Kontakte **vor neun Tagen** an, an die Sie sich konkret erinnern können.

Um welchen Wochentag handelte es sich?

[Bitte auswählen]

Relevant sind nur Kontakte auf die **mindestens eins der folgenden Kriterien zutrifft**:

Menschen
- mit denen Sie ein **Gespräch** geführt haben (Abstand < 1,5m)
- mit denen Sie **mehr als 10 Minuten engen Kontakt** hatten (Abstand < 1,5m)
- mit denen Sie **mehr als 10 Minuten in einem geschlossenen Raum** verbracht haben

Sollten Sie keine Kontakte einer Kategorie gehabt haben, tragen Sie bitte eine **0** ein.

Hier können Sie sich die Kategorien erneut ansehen, wenn Sie unsicher sind.

	Anzahl erinnerter und namentlich bekannter Kontaktpersonen
Haushaltsmitglied	
Familie / Freunde	
Arbeits- / Schulkontakte	
Medizinische Versorgung (z.B. Arztbesuch)	
Öffentliche Verkehrsmittel	
Einkaufen	
Freizeitaktivitäten (z.B. Sport, Kinobesuch)	
Andere	

Zurück Weiter

JUSTUS-LIEBIG-UNIVERSITÄT GIESSEN

66% ausgefüllt

Bitte geben Sie nun die Anzahl relevanter Kontakte **vor zehn Tagen** an, an die Sie sich konkret erinnern können.

Um welchen Wochentag handelte es sich?

[Bitte auswählen]

Relevant sind nur Kontakte auf die **mindestens eins der folgenden Kriterien zutrifft**:

Menschen
- mit denen Sie ein **Gespräch** geführt haben (Abstand < 1,5m)
- mit denen Sie **mehr als 10 Minuten engen Kontakt** hatten (Abstand < 1,5m)
- mit denen Sie **mehr als 10 Minuten in einem geschlossenen Raum** verbracht haben

Sollten Sie keine Kontakte einer Kategorie gehabt haben, tragen Sie bitte eine **0** ein.

Hier können Sie sich die Kategorien erneut ansehen, wenn Sie unsicher sind.

	Anzahl erinnerter und namentlich bekannter Kontaktpersonen
Haushaltsmitglied	
Familie / Freunde	
Arbeits- / Schulkontakte	
Medizinische Versorgung (z.B. Arztbesuch)	
Öffentliche Verkehrsmittel	
Einkaufen	
Freizeitaktivitäten (z.B. Sport, Kinobesuch)	
Andere	

Zurück

Weiter

JUSTUS-LIEBIG-UNIVERSITÄT GIESSEN

71% ausgefüllt

Bitte geben Sie nun die Anzahl relevanter Kontakte **vor elf Tagen** an, an die Sie sich konkret erinnern können.

Um welchen Wochentag handelte es sich?

[Bitte auswählen]

Relevant sind nur Kontakte auf die **mindestens eins der folgenden Kriterien zutrifft**:

Menschen
- mit denen Sie ein **Gespräch** geführt haben (Abstand < 1,5m)
- mit denen Sie **mehr als 10 Minuten engen Kontakt** hatten (Abstand < 1,5m)
- mit denen Sie **mehr als 10 Minuten in einem geschlossenen Raum** verbracht haben

Sollten Sie keine Kontakte einer Kategorie gehabt haben, tragen Sie bitte eine **0** ein.

Hier können Sie sich die Kategorien erneut ansehen, wenn Sie unsicher sind.

	Anzahl erinnerter und namentlich bekannter Kontaktpersonen
Haushaltsmitglied	
Familie / Freunde	
Arbeits- / Schulkontakte	
Medizinische Versorgung (z.B. Arztbesuch)	
Öffentliche Verkehrsmittel	
Einkaufen	
Freizeitaktivitäten (z.B. Sport, Kinobesuch)	
Andere	

Zurück

Weiter

JUSTUS-LIEBIG-UNIVERSITÄT GIESSEN

75% ausgefüllt

Bitte geben Sie nun die Anzahl relevanter Kontakte **vor zwölf Tagen** an, an die Sie sich konkret erinnern können.

Um welchen Wochentag handelte es sich?

[Bitte auswählen]

Relevant sind nur Kontakte auf die **mindestens eins der folgenden Kriterien zutrifft**:

Menschen
- mit denen Sie ein **Gespräch** geführt haben (Abstand < 1,5m)
- mit denen Sie **mehr als 10 Minuten engen Kontakt** hatten (Abstand < 1,5m)
- mit denen Sie **mehr als 10 Minuten in einem geschlossenen Raum** verbracht haben

Sollten Sie keine Kontakte einer Kategorie gehabt haben, tragen Sie bitte eine **0** ein.

Hier können Sie sich die Kategorien erneut ansehen, wenn Sie unsicher sind.

	Anzahl erinnerter und namentlich bekannter Kontaktpersonen
Haushaltsmitglied	
Familie / Freunde	
Arbeits- / Schulkontakte	
Medizinische Versorgung (z.B. Arztbesuch)	
Öffentliche Verkehrsmittel	
Einkaufen	
Freizeitaktivitäten (z.B. Sport, Kinobesuch)	
Andere	

Zurück Weiter

JUSTUS-LIEBIG-UNIVERSITÄT GIESSEN

79% ausgefüllt

Bitte geben Sie nun die Anzahl relevanter Kontakte **vor 13 Tagen** an, an die Sie sich konkret erinnern können.

Um welchen Wochentag handelte es sich?

[Bitte auswählen]

Relevant sind nur Kontakte auf die **mindestens eins der folgenden Kriterien zutrifft**:

Menschen
- mit denen Sie ein **Gespräch** geführt haben (Abstand < 1,5m)
- mit denen Sie **mehr als 10 Minuten engen Kontakt** hatten (Abstand < 1,5m)
- mit denen Sie **mehr als 10 Minuten in einem geschlossenen Raum** verbracht haben

Sollten Sie keine Kontakte einer Kategorie gehabt haben, tragen Sie bitte eine **0** ein.

Hier können Sie sich die Kategorien erneut ansehen, wenn Sie unsicher sind.

	**Anzahl erinnerter und namentlich bekannter Kontaktpersonen**
Haushaltsmitglied	
Familie / Freunde	
Arbeits- / Schulkontakte	
Medizinische Versorgung (z.B. Arztbesuch)	
Öffentliche Verkehrsmittel	
Einkaufen	
Freizeitaktivitäten (z.B. Sport, Kinobesuch)	
Andere	

Zurück

Weiter

JUSTUS-LIEBIG-UNIVERSITÄT GIESSEN

83% ausgefüllt

Bitte geben Sie nun die Anzahl relevanter Kontakte **vor 14 Tagen** an, an die Sie sich konkret erinnern können.

Um welchen Wochentag handelte es sich?

[Bitte auswählen]

Relevant sind nur Kontakte auf die **mindestens eins der folgenden Kriterien zutrifft**:

Menschen
- mit denen Sie ein **Gespräch** geführt haben (Abstand < 1,5m)
- mit denen Sie **mehr als 10 Minuten engen Kontakt** hatten (Abstand < 1,5m)
- mit denen Sie **mehr als 10 Minuten in einem geschlossenen Raum** verbracht haben

Sollten Sie keine Kontakte einer Kategorie gehabt haben, tragen Sie bitte eine **0** ein.

Hier können Sie sich die Kategorien erneut ansehen, wenn Sie unsicher sind.

	Anzahl erinnerter und namentlich bekannter Kontaktpersonen
Haushaltsmitglied	
Familie / Freunde	
Arbeits- / Schulkontakte	
Medizinische Versorgung (z.B. Arztbesuch)	
Öffentliche Verkehrsmittel	
Einkaufen	
Freizeitaktivitäten (z.B. Sport, Kinobesuch)	
Andere	

Zurück Weiter

JUSTUS-LIEBIG-UNIVERSITÄT GIESSEN

87% ausgefüllt

Für wie viele Tage haben Sie Hilfsmittel (z.B. Terminkalender) zur Ermittlung von Kontakten genutzt?

[Bitte auswählen]

Zurück

Weiter

JUSTUS-LIEBIG-
UNIVERSITÄT
GIESSEN

92% ausgefüllt

Vielen Dank, dass Sie unseren Fragebogen ausgefüllt haben!

Für unsere Forschung ist es sehr wichtig zu wissen, ob Sie diesen Fragebogen gewissenhaft ausgefüllt haben. Sie erhalten Ihre Vergütung unabhängig davon, wie Sie die folgende Frage beantworten, also bitte antworten Sie ehrlich. **Diese Information kann nicht auf Sie zurückgeführt werden und hat keinen Einfluss auf Ihre Vergütung.**

Ich habe den Fragebogen gewissenhaft ausgefüllt

○ ja

○ nein

Zurück

Weiter
